# Estimation of Stature From Percutaneous Palpable Measurements of Radius, Ulna, and Humerus Among South Indian Undergraduate Students: A Regression Analysis

**DOI:** 10.7759/cureus.113588

**Published:** 2026-07-29

**Authors:** Sikander Quddusi, P. Moula Akbar Basha, Aastha Mohale

**Affiliations:** 1 Department of Anatomy, Fathima Institute of Medical Sciences, Kadapa, IND; 2 Department of Anatomy, All India Institute of Medical Sciences, Rishikesh, IND

**Keywords:** anatomy, humerus, radius, regression analysis, stature estimation, ulna

## Abstract

Introduction

Estimation of stature from skeletal remains is a fundamental aspect of forensic anthropology and medico-legal investigations. Upper limb long bones, i.e., humerus, radius, and ulna, are commonly used for this purpose due to their strong correlation with body height. However, regression models are population-specific, necessitating region-based studies. The present study aims to estimate stature from upper limb bone lengths in an adult male population of Andhra Pradesh, South India.

Materials and methods

A cross-sectional study was conducted on 500 adult male subjects aged between 20 and 35 years in the South Indian population. Percutaneous measurements of the humerus, radius, and ulna were taken on the right side and left side using a standard measuring tape. Stature was measured using a stadiometer. Statistical analysis included calculation of mean, standard deviation, and correlation coefficients. Multiple linear regression analysis was performed to derive an equation for stature estimation. Analysis of variance (ANOVA) was used to assess the significance of the regression model.

Results

Multiple linear regression analysis demonstrated that the overall model was statistically significant (F = 219.275, p < 0.001). The model explained 72.7% of the variance in stature (R² = 0.727). Among the six predictors, right ulna length (B = 2.227, p < 0.001), left humerus length (B = 1.549, p < 0.001), and left radius length (B = 1.256, p < 0.001) were significant independent predictors of stature. In contrast, right humerus, right radius, and left ulna did not contribute significantly to the model after adjustment (p > 0.05). Multiple linear regression analysis demonstrated that the overall model was statistically significant (F = 219.275, p < 0.001). The model explained 72.7% of the variance in stature (R² = 0.727). Among the six predictors, right ulna length (B = 2.227, p < 0.001), left humerus length (B = 1.549, p < 0.001), and left radius length (B = 1.256, p < 0.001) were significant independent predictors of stature. In contrast, right humerus, right radius, and left ulna did not contribute significantly to the model after adjustment (p > 0.05).

Conclusions

Upper limb bones are a reliable predictor of stature, with the ulna being the most reliable bone for stature estimation in the male population of Andhra Pradesh. The regression equation derived in this study can be effectively used in forensic identification and anthropological assessments within similar populations.

## Introduction

Skeletal remains are often encountered in situations involving mass burials, commingled remains, or surface scattering following mass disasters, criminal activities, or events causing multiple fatalities, such as terrorist attacks. Estimation of stature from skeletal elements plays a crucial role in narrowing the biological profile of unidentified individuals in forensic investigations. In such scenarios, the role of the forensic anthropologist becomes critical in accurately analyzing, sorting, and attributing skeletal elements to the appropriate individuals [1]. Human identification of unknown remains is frequently accomplished using long bones, as they provide reliable parameters for estimating stature through established anthropometric methods [2]. The challenge of correlating long bone lengths with body height has long occupied scientists, who have proposed several formulas to estimate the height of various demographic groups using different skeletal components and techniques [3].

One of the most reliable metrics for determining height is arm span [4]. Although numerous formulas have been developed for estimating height from long bone lengths, their application across different populations remains challenging [5]. For instance, the formulae developed for the African population are inappropriate for the Asian population, as African individuals have comparatively longer arms and legs [6]. Similarly, the diverse geographic and population variations in a country like India make it necessary to develop population-specific formulae for different regions.

In the Kadapa region of Andhra Pradesh, there has been an increase in events leading to retrieval of unidentified bodies, including mass casualties, natural disasters (such as Cyclone Nivar in November 2020), accidents, or even homicides [7]. However, a regression equation in this population is currently lacking. Therefore, the present study aimed to develop a regression equation to estimate stature from upper limb bone lengths of the male population in the Kadapa district of Andhra Pradesh.

## Materials and methods

Study design

A cross-sectional, observational study was carried out in 500 male students aged between 20 and 35 years. The study participants included medical students of Fathima Institute of Medical Sciences, Kadapa, Andhra Pradesh, enrolled between January 2025 and December 2025. Ethical approval was obtained from the Institutional Review Committee vide letter number IEC/FIMS - M021/Acad/IEC No. 039/2025-26, and informed written consent was obtained from all participants. The study participants comprised the usual mix of individuals residing in this region (Kadapa district), having migrated from Andhra Pradesh (n = 202), Kerala (n = 115), Tamil Nadu (n = 104), and Karnataka (n = 79), thus representing the study population. Those with prior nutritional deficiencies, musculoskeletal, congenital, or acquired deformities, or a history of upper limb amputation were excluded from the study.

Anthropometric measurement

Anthropometric measurements were obtained from the study participants by a single observer from the Department of Anatomy, Fathima Institute of Medical Sciences, Kadapa, Andhra Pradesh, to reduce inter-observer variability. Stature was measured with subjects standing erect in the standard anatomical position, barefoot, and with the head in the Frankfort horizontal plane. Height was recorded from vertex to heel using a stadiometer (item code: BO6VT3ZLR5, Prime Surgicals, India).

Percutaneous lengths of the humerus, radius, and ulna were measured using a fiberglass body measuring tape (Gemsy, India; accuracy verified against the stadiometer to ensure precision), based on defined anatomical landmarks. Humeral length was measured from the greater tubercle of the humerus to the lateral epicondyle with the elbow flexed over the chest. Radial length was measured from the head of the radius to the radial styloid process, and ulnar length from the olecranon process to the ulnar styloid process, both with the elbow flexed. All measurements were recorded in centimeters, with the participant sitting on a chair.

Statistical analysis

Data on humeral, radial, and ulnar lengths, along with corresponding stature measurements, were analyzed using SPSS version 27 (IBM Corp., Armonk, NY). Descriptive statistics including mean, standard deviation, and range were computed for all variables using Microsoft Office Excel 2021 (Microsoft Corp., Redmond, WA). The relationship between stature and the lengths of the humerus, radius, and ulna was evaluated using the Pearson correlation coefficient. Linear regression analysis was performed to determine the extent to which humeral, radial, and ulnar lengths could predict stature. A p-value of <0.05 was considered statistically significant.

## Results

During the study period from January 2025 to December 2025, a total of 500 healthy male participants aged 20-35 years were included. The mean stature of the study population was 173.50 ± 10.98 cm. The mean right humeral, radial, and ulnar lengths were 32.21 ± 2.37 cm, 26.57 ± 1.68 cm, and 27.78 ± 1.75 cm, respectively. Corresponding left-sided measurements were 32.28 ± 2.21 cm, 26.57 ± 1.76 cm, and 27.75 ± 1.77 cm. The mean values on both sides were comparable, indicating bilateral symmetry of upper limb measurements in the study population. Table 1 presents the mean length and standard deviation for the humerus, radius, and ulna of both sides.

**Table 1:** Descriptive statistics of height and bony length of upper limb in male participants

Variable	Sample	Mean	Standard deviation
Humerus right side	500	32.21	2.37
Radius right side	500	26.57	1.68
Ulna right side	500	27.78	1.75
Humerus left side	500	32.28	2.21
Radius left side	500	26.57	1.76
Ulna left side	500	27.75	1.77
Height	500	173.5	10.98

Table 2 shows the correlation matrix, indicating that the ulna and humerus showed higher correlation with stature than the radius. All upper limb bone measurements demonstrated a statistically significant positive correlation with stature (p < 0.01). The right ulna showed the strongest correlation with stature (r = 0.82), followed by the right humerus (r = 0.66) and left radius (r = 0.75). The left ulna (r = 0.72) also exhibited a strong positive association. These findings indicate that increasing upper limb bone length is associated with increased stature, with the ulna serving as the strongest individual predictor.

**Table 2:** Correlation matrix

Side of the participant	r value	p-value
Humerus right side	0.66	≤0.01
Radius right side	0.79	≤0.01
Ulna right side	0.82	≤0.01
Humerus left side	0.79	≤0.01
Radius left side	0.75	≤0.01
Ulna left side	0.72	≤0.01

Multiple linear regression analysis demonstrated a strong relationship between upper limb bone lengths and stature. The regression model showed the coefficient of determination of R2 = 0.724, indicating that approximately 72% of the variation in stature could be explained by the included upper limb measurements. The standard error of estimate was 5.77 cm, indicating acceptable prediction accuracy (Table 3).

**Table 3:** Multivariable linear regression model for stature estimation using right-sided upper limb bone lengths

Model	R	R^2^	Adjusted R^2^	Standard error of the estimate
1	0.834	0.72	0.72	5.77
R square change	F change	df1	df2	Significance of F change
0.727	219.3	6	493	<0.001

Analysis of variance (ANOVA) demonstrated that the overall regression model was highly statistically significant (F = 219.275, p < 0.001), indicating that the combination of upper limb bone lengths significantly predicts stature and that the regression equation provides a better fit than a model without predictor variables (Table 4).

**Table 4:** Analysis of variance

Model		Sum of squares	df	Mean square	F	Significance
1	Regression	43793.72	6	7298.95	219.275	
	Residual	16410.34	493	33.287		
	Total	60204.05	499			

Multiple linear regression analysis (Table 5) demonstrated that the overall model was statistically significant (F = 219.275, p < 0.001). The model explained 72.7% of the variance in stature (R² = 0.727). Among the six predictors, right ulna length (B = 2.227, p < 0.001), left humerus length (B = 1.549, p < 0.001), and left radius length (B = 1.256, p < 0.001) were significant independent predictors of stature. In contrast, right humerus, right radius, and left ulna did not contribute significantly to the model after adjustment (p > 0.05).

**Table 5:** Regression coefficient analysis

	Unstandardized coefficients			t	Significance	95% confidence interval for B	
Model t	B	Standard error	Beta			Lower bound	Upper bound
Constant	21.018	4.344	aaaa	4.838	0	12.483	29.553
Length of humerus right side	0.239	0.174	0.052	1.374	0.17	-0.103	0.581
Length of radius right side	0.029	0.452	0.004	0.064	0.949	-0.859	0.917
Length of ulna right side	2.227	0.478	0.356	4.656	0	1.287	3.167
Length of humerus left side	1.549	0.239	0.312	6.489	0	1.08	2.018
Length of radius left side	1.256	0.29	0.201	4.337	0	0.687	1.826
Length of ulna left side	-0.044	0.292	-0.007	-0.15	0.88	-0.617	0.53

## Discussion

The present study evaluated the relationship between upper limb long bone lengths and stature in an adult male population from South India. The findings demonstrate a statistically significant and strong positive correlation between stature and the lengths of the humerus, radius, and ulna, supporting their utility in forensic and anthropometric applications. In the current study, all measured bones exhibited significant correlation coefficients (r = 0.724), indicating a strong linear association with height. Among these, the ulna showed the highest correlation, followed by the humerus and radius. Similar findings have been reported in Indian and South Asian populations, where the ulna has been identified as the most dependable predictor among upper limb bones [4,8-12].

Pearson correlation analysis revealed strong positive associations between stature and all upper limb bone lengths. Among these, left ulna demonstrated the highest correlation coefficient, indicating that it is the most reliable single predictor of stature in the present population. The relatively higher correlation observed for the ulna may be attributed to its comparatively stable anatomical growth pattern and lower susceptibility to soft tissue variation during percutaneous measurements. The multiple regression model demonstrated satisfactory predictive ability for estimating stature using upper limb bone measurements. An R² value of 0.724 indicates that nearly 60% of the variation in stature is explained by the three measured bones. Although factors such as genetics, nutrition, and environmental influences contribute to the remaining unexplained variation, the present model provides reliable predictive accuracy for forensic applications.

The highly significant ANOVA result confirms that the regression equation possesses strong overall predictive validity. The observed F-statistic indicates that the combined contribution of humeral, radial, and ulnar lengths significantly improves stature prediction compared with random variation alone. This finding strengthens the reliability of the proposed regression equation for forensic anthropological practice.

Regression coefficient analysis demonstrates that not all upper limb measurements contribute equally when evaluated simultaneously. The right ulna emerged as the strongest independent predictor, emphasizing its value for stature estimation. The lack of significance for certain variables after multivariable adjustment is likely due to shared variance (multicollinearity) among upper limb measurements rather than a lack of biological association. Because humerus, radius, and ulna lengths are themselves highly correlated, some predictors lose statistical significance when entered into the same regression model.

Hariharan et al. [8] percutaneously assessed right ulnar length across both sexes and formulated linear regression equations. Their results revealed a direct association between ulnar length and stature, with ulnar length being greater in men than in women.

Mehta et al. [9] conducted a study on 100 participants aged 18-30 years from the Bhopal region. The Pearson correlation coefficient for the right ulna was r = 0.754 and for the left ulna r = 0.70, both indicating a statistically significant correlation between stature and ulnar length.

Pandey et al. [10] conducted a study on 300 male and female participants aged 18-28 years from Bangalore. Ulnar length on both sides was correlated with stature. For male participants, r = 0.45 (right) and r = 0.51 (left); for female participants, r = 0.27 (right) and r = 0.32 (left). A statistically significant correlation was found between stature and ulnar length in all groups.

Chandragirish et al. [13] recruited 200 participants aged between 18 and 30 years from Karnataka to investigate the association between humeral length and stature. Their findings demonstrated a statistically significant relationship between humeral length and stature in both sexes. The standardized coefficient (Beta) was 0.650 for male participants and 0.454 for female participants.

Ijaz et al. [14] enrolled 100 male participants to examine the relationship between stature and upper limb length using Pearson correlation. Wingspan showed the highest correlation coefficient, suggesting it to be the most reliable single predictor of stature. Linear regression analysis further showed that wingspan and brachial lengths on both sides could accurately predict stature.

Supare et al. [15] recruited 400 participants between 18 and 24 years of age from Maharashtra to investigate the association between stature and arm span. A statistically significant correlation was documented in both sexes (R = 0.89 in male participants; R = 0.90 in female participants). The authors inferred that arm span reliably predicts stature and may serve as a practical substitute in individuals with physical deformity or mutilated skeletal remains. A brief comparison of key findings from other previous studies is summarized in Table 6.

**Table 6:** Review of published literature comparing regression analysis in different studies r: correlation coefficient, R2: coefficient of determination, Hum: humerus, Rad: radius

Author	Region	Age	Parameter used	R2 coefficient of determination	Findings
Sarma et al. (2022) [4]	North East India	25-45 years	Right and left ulnar bone length in males and females	Male: r = 0.915, female: r = 0.836	Ulnar length provided more accurate means in estimating the height of the individual
Narayan et al. (2025) [11]	Bihar, India	18-65 years	Right and left long bones of upper limb	Hum: r = 0.56, rad: r = 0.54, ulna: r = 0.64	Ulna bone showed the highest correlation
Panchal et al. (2025) [12]	Gujarat, India	20-50 years	Right and left long bones of upper limb in males and females	Hum: r = 0.78, rad: r = 0.70, ulna: r = 0.84	Ulna showed the highest correlation
Ahmed (2013) [16]	Sudanese Arab	25-30 years	Upper arm, ulna bone, wrist breadth, hand length and breadth	Males: r = 0.725, females: r = 0.722	Ulnar bone length was the most suitable predictor for stature
Uzun et al. (2020) [17]	Turkey	20-27 years	Arm span	Males: r = 0.785, females: r = 0.807	Arm span dependable parameter for estimating stature
Mahakkanukrauh et al. (2011) [18]	Thailand	Skeleton of 67 years	Humerus, radius, and ulna for both sexes	Males: Hum: r = 0.667, rad: r = 0.672, ulna: r = 0.660; females: hum: r = 0.552, rad: r = 0.616, ulna: r = 0.567	Ulnar length provided more accuracy in estimating the height of an individual

Limitations

The study has some limitations. Females were not included owing to logistical constraints. There is a well-established difference in stature and bone length between males and females, and including both sexes would necessitate separate regression analyses for each. The derived equation is therefore applicable to males only; a separate study would be required to derive a regression-based formula for females. The study did not check inter-observer reproducibility by involving measurements from multiple observers; the equation was evaluated only in-sample (no split-sample or cross-validation), and no radiological assessment could be performed to assess the accuracy of the measurements by percutaneous method.

## Conclusions

The three upper limb long bones (the humerus, radius, and ulna) are reliable predictors of stature in the male population of Andhra Pradesh. Among these, the ulna showed the highest correlation with stature, followed by the humerus and radius. For optimal predictive accuracy, the combined use of all three bones is recommended where feasible. The derived regression equation can serve as a valuable tool in forensic identification and anthropological research. Future studies should focus on deriving a comparable regression equation for females from this region.
